# The capsid proteins of orthoflavivirus are interchangeable for West Nile virus assembly

**DOI:** 10.1128/jvi.01227-26

**Published:** 2026-09-25

**Authors:** Jing Wang, Bao-Di Shang, Cheng-Lin Deng, Xiao-Dan Li, Bo Zhang

**Affiliations:** 1 State Key Laboratory of Virology and Biosafety, Wuhan Institute of Virology, Chinese Academy of Sciences 74614, Wuhan, China; 2 University of Chinese Academy of Sciences 74519, Beijing, China; 3 Guizhou Fisheries Research Institute 650994, Guiyang, China; 4 School of Medicine, Hunan Normal University 117930, Changsha, China; 5 Hubei Jiangxia Laboratory 854985 , Wuhan, China; The University of Texas Southwestern Medical Center, Dallas, Texas, USA

**Keywords:** orthoflavivirus, West Nile virus, dengue virus, capsid protein, encapsidation

## Abstract

Orthoflavivirus virions consist of an envelope enclosing a nucleocapsid core. A critical step in orthoflavivirus assembly is the encapsidation of genomic RNA by the capsid protein to form the nucleocapsid. However, the molecular details of this process remain poorly understood, particularly whether capsid proteins bind genomic RNA through specific interactions. Here, we utilized West Nile virus (WNV) and dengue virus (DENV) as model systems to investigate heterologous genome RNA encapsidation. Employing a chimeric virus strategy, we demonstrated that the DENV-2 capsid protein can package WNV genomic RNA if two critical elements are preserved: *cis*-acting sequences essential for RNA synthesis and the cleavage site required for capsid maturation. Furthermore, by decoupling the WNV *cis*-acting elements from the capsid-coding sequence and replacing the WNV capsid protein with orthologous capsid proteins from other orthoflaviviruses, we provide direct virological evidence that orthoflavivirus capsid proteins package genomic RNAs in a non-sequence-specific manner. These findings advance our mechanistic understanding of orthoflavivirus genome packaging.

**IMPORTANCE:**

The encapsidation of genomic RNA by capsid proteins as the primary event in orthoflavivirus virion assembly is not well understood in current research; particularly, whether it involves specific packaging signals remains a question. The orthoflavivirus capsid protein plays a key role in both genome replication and viral particle assembly. Our comprehensive virological analyses reveal that orthoflavivirus capsid protein shows considerable interspecies packaging flexibility while critical replication and processing elements are preserved. These insights underscore a key aspect of orthoflavivirus biology: the conserved packaging function of capsid proteins across genus members is mediated by structural interactions, rather than sequence specificity.

## INTRODUCTION

The genus *Orthoflavivirus* comprises over 70 viruses, including clinically significant human pathogens such as West Nile virus (WNV), Japanese encephalitis virus (JEV), Zika virus (ZIKV), dengue virus (DENV), yellow fever virus (YFV), and tick-borne encephalitis virus (TBEV) (1). Among these, DENV is globally dominant: its four serotypes (DENV-1 to DENV-4) cause an estimated 390 million infections annually (2). Elucidating orthoflavivirus life cycles, especially the understudied mechanisms of genome encapsidation and virion assembly, is critical for developing effective prevention and therapeutic strategies (3).

Orthoflaviviruses are ~50 nm in diameter and harbor a positive-sense, single-stranded RNA genome (~11 kb) with a 5′ cap structure [but no 3′ poly(A) tail] and highly structured 5′/3′ untranslated regions (UTRs) that regulate viral replication (4). A single open reading frame encodes a polyprotein, which is processed into three structural proteins (C, prM, and E) required for virion assembly (5, 6) and seven non-structural proteins (NS1–NS5) involved in RNA replication, polyprotein processing, and host immune modulation (7). Orthoflavivirus RNA synthesis depends on genome cyclization, mediated by long-range RNA–RNA interactions between complementary sequences in the 5′ and 3′ (8, 9). Key *cis*-acting elements in the 5′ UTR and capsid regions facilitate genome cyclization through sequence-specific base pairing with their 3′ counterparts (10–12). Additionally, structural motifs such as the SLA, SLB, and 5′-UAR-flanking stem (UFS) recruit the NS5 RNA-dependent polymerase to the 5′ end of the genome, ensuring efficient RNA synthesis (13, 14). Collectively, these conserved RNA elements orchestrate viral RNA replication.

Orthoflavivirus assembly is tightly coupled to RNA replication (15, 16), which occurs at the endoplasmic reticulum membrane. A key step in assembly is the interaction between genomic RNA and the capsid protein to form nucleocapsid cores (NCs), a prerequisite for subsequent virion assembly (17). Despite their importance, NC structures remain unresolved in mature orthoflaviviruses by cryo-EM (18). The capsid protein, initially cytosolic, undergoes NS2B/3 protease-mediated cleavage at the capsid–anchor junction to achieve functional maturation (19). Although orthoflavivirus capsid proteins share low sequence homology, they exhibit high structural conservation (3). Structural studies of JEV, DENV, TBEV, and ZIKV capsids have identified two conserved interfaces critical for assembly: (i) the hydrophobic α2-α2′ surface, which mediates membrane association, and (ii) the positively charged α4-α4′ surface, which facilitates RNA binding (20–23). The current understanding of nucleocapsid assembly in orthoflaviviruses is largely derived from *in vitro* studies. In such systems, the DENV capsid protein has been shown to assemble nucleocapsid-like particles (NLPs) with positive-sense DENV genomic RNAs of varying lengths. Notably, the capsid protein also forms similar NLPs with negative-sense DENV RNA, indicating that the DENV capsid protein has no specificity toward the positive or negative strands of DENV RNA. Likewise, the ZIKV capsid protein exhibits a broad binding mode to different nucleotide types, including single-stranded and double-stranded RNA and DNA. These observations support a model in which initial RNA binding is mediated by non-specific electrostatic interactions, a process facilitated by the spatial co-localization of viral components at assembly sites (17, 24).

However, during the viral life cycle, the capsid appears to exhibit some degree of sequence specificity in packaging the genomic RNA. For example, certain chimeric C-prM-E constructs fail to package TBEV subgenomic replicon RNAs into virus-like particles (VLPs), despite proper structural protein processing (25), implying that homologous interaction between C and non-structural proteins or the genomic RNA is required for efficient assembly. Additionally, NS2A, a putative assembly factor that binds the 3′ UTR, may mediate packaging specificity (26, 27). Orthoflavivirus genomic RNA has not been reported to contain a bona fide packaging signal that enables specific binding and packaging by the capsid protein. Moreover, the detailed molecular interactions involved and their effects on orthoflavivirus particle assembly remain obscure. Critical questions remain: during infectious virion packaging, do orthoflavivirus capsid proteins selectively package only homologous genomic RNAs? Or can they package heterologous genomic RNAs? Are there conserved structural motifs or sequence elements that function as “packaging signals” that ensure selective RNA incorporation?

To address these questions, we used a chimeric virus approach to systematically investigate orthoflavivirus capsid-mediated RNA packaging specificity. We engineered recombinant WNV carrying heterologous DENV-2 capsid proteins (chosen for their approximately 35% amino acid identity with the WNV capsid, maximizing sequence divergence) to directly assess packaging efficiency and specificity in the context of native viral replication and assembly. Our results demonstrate that orthoflavivirus capsid proteins can effectively package heterologous genomic RNAs, providing direct evidence for non-sequence-specific packaging across the genus.

## MATERIALS AND METHODS

### Cell culture and antibodies

BHK-21 and HEK293T cells were cultured in Dulbecco’s modified Eagle’s medium (DMEM, Gibco). The mosquito C6/36 cells were grown in RPMI 1640 medium (Gibco). All media were supplemented with 10% (vol/vol) fetal bovine serum (FBS, Sigma-Aldrich), penicillin (100 U/mL)–streptomycin (100 μg/mL) liquid (Solarbio). BHK-21 and HEK293T cells were maintained in 5% CO_2_ at 37°C, while C6/36 cells were maintained at 28°C. The antibodies used in our study included rabbit anti-WNV-NS2B/3 polyclonal antibody (prepared by immunizing rabbits with purified proteins), mice anti-ZIKV-C polyclonal antibody (prepared by immunizing mice with purified proteins), mice anti-DENV-C antibody (GeneTex), rabbits anti-WNV-E and anti-WNV-prM antibodies (GeneTex), anti-His and anti-HA monoclonal antibodies (TIANGEN), goat antimouse IgG polyclonal antibody conjugated with Alexa Fluor 488 (Abcam), goat antirabbit IgG polyclonal antibody conjugated with Alexa Fluor 568 (Abcam), and goat antimouse and antirabbit IgG polyclonal antibodies conjugated with horseradish peroxidase (Proteintech).

### Plasmid construction

Two infectious clones of WNV WT (strain NY99) (28) and DENV-2 WT (Strain D2Y98P) (29) were utilized to construct the capsid chimeras. A *Renilla* luciferase (Rluc) reporter replicon, WNV-rep-DV2mC, was constructed by replacing the WNV capsid gene with the DENV-2 mature capsid gene in frame through PCR using the WNV-Rluc-2A plasmid as the backbone. To construct the infectious clones of WNV-C57(T143C)-FL and WNV-C57-FL(T143C) mutants, fragments with the desired 5′CS mutation were engineered into the C57-derived fragment or the full-length capsid-coding region.

The WNV-C57 infectious clone was constructed based on the WNV WT backbone. Briefly, two fragments covering BamHI-T7-WNV 5′ UTR-C57 (aa) -2A and 2A-C-prME-EcoNI were amplified, fused together by overlap PCR, and inserted into WNV WT to produce the WNV-C57 infectious clone. Similarly, BamHI-T7-WNV 5′ UTR-C57 (aa) -2A-C-prME-EcoNI fragments containing mature capsid substitution from DENV-1 strain (GenBank accession NC_001477), DENV-2, ZIKV strain SZ-WIV01 (GenBank accession no. KU963796), YFV-17D strain (GenBank accession MN708488), and TBEV (GenBank accession NC_001672) were amplified, respectively. These fragments were used to replace the corresponding sequences of WNV WT, generating the infectious clones WNV-C57-DV1mC, WNV-C57-DV2mC, WNV-C57-ZIKVmC, WNV-C57-YFVmC, and WNV-C57-TBEVmC.

WNV-NS2B/3, WNV-Cpr, and chimeric Cpr-encoding constructs were obtained by cloning the sequence of interest into the pCAGGS expression vector. The WNV-NS2B/3 construct was fused with an HA tag at the C-terminus, while the Cpr constructs were fused with a His tag at the N-terminus. The plasmids for single-round infectious particle (SRIP) production were constructed using a VEEV replicon vector system to express the mature capsid protein and prME protein separately. The corresponding gene fragments were inserted into VEEV vector through AscI and PacI restriction sites. All plasmids were validated by DNA sequencing before the subsequent experiments.

### *In vitro* RNA transcription

After linearization and purification with phenol-chloroform, viral genomic RNAs were transcribed using a mMessage mMachine T7 Transcription Kit (Invitrogen, USA) according to the manufacturer's protocols. The transcribed RNAs were quantified by spectrophotometry and analyzed by agarose gel electrophoresis to examine the quality.

### Virus recovery

The viral RNAs were transfected into BHK-21 cells with DMRIE-C (Invitrogen) according to the manufacturer’s instructions. At different time points post-transfection, the supernatants containing viruses were collected and stored as aliquots at −80°C for the following experiments. To obtain replication-competent viruses of WNV-DV2C and WNV-DV2mC, chimeras were subjected to serial passages in C6/36 cells at 28°C. Total RNAs of WNV-DV2mC-P10-A/B/C-infected cells were extracted, and RT-PCR was then carried out using the PrimeScript One Step RT-PCR Kit (Takara) with primers to amplify the fragments spanning from 5′ UTR to 3′ UTR. The products were sequenced to characterize the genetic changes in the viral genome.

### Production of SRIPs

BHK-21 cells were co-transfected with three RNAs, 1 μg of WNV-Rluc2A replicon RNA, 1 μg of mature capsid-expression (VEEV-WNV/DV1/DV2/ZIKV/YFV/TBEV-mC) RNA, and 1 μg of WNV prME-expression (VEEV-WNV-prME) RNA, using DMRIE-C transfection reagent. The supernatant was collected at 36/48 hpt, and 200 μL of the supernatant was used to infect Vero cells. The infected cells were lysed at 24 hpi and used for measuring luciferase activity.

### Luciferase assay

The RNAs (5 μg) of WNV-rep-DV2mC and its mutants were electroporated into 8 × 10^6^ BHK-21 cells as described previously (30). The cells were seeded in 12-well plates, and cell lysates were collected at different time points. Luciferase activity was measured using a microplate reader (Varioskan Flash; Thermo Fisher Scientific, Finland) after mixing 20 μL of the lysates with 50 μL of substrate (Promega).

### Immunofluorescence assay

Cells were seeded onto Chamber Slides (Nalge Nunc) in 35 mm dishes. At given time points, the cells were fixed with pre-cooled (−20°C) 5% acetone in methanol for 10 min and then washed three times with PBS. The slides were then incubated with primary antibody at room temperature for 1 h. Following three washes with PBS, the slides were further incubated with secondary antibody for an additional hour. Finally, the slides were stained with DAPI reagent and mounted with 95% glycerol. Fluorescence data were collected and analyzed under a Zeiss fluorescence microscope.

### Plaque assay

The quantification of infectious virus particles was performed using plaque assay as previously described (31). The titer and plaque morphology of WNV-DV2mC and the WNV-C57-mC chimeric viruses were determined using double-layer plaque assay and monolayer plaque assay, respectively. Briefly, for the double-layer plaque assay, BHK-21 cells (1.5 × 10^5^ per well) were seeded into 12-well plates. After 1 day of cultivation, the cells were incubated with 10-fold diluted viruses at 37°C for 1 h before the first layer of agar was added. A second layer of agar containing neutral red was added after 3 days of incubation at 37°C with 5% CO_2_. Plaque morphology and numbers were recorded after incubating the plates for an additional 12–24 h. For monolayer plaque assay, BHK-21 cells (1 × 10^5^ per well) were seeded into 24-well plates. On the following day, the cells were infected with 10-fold diluted viruses and overlaid with a DMEM semisolid medium containing 1% (wt/vol) methylcellulose, 2% FBS, and penicillin (100 U/mL)–streptomycin (100 μg/mL). The plates were incubated at 37°C with 5% CO_2_ for 3 days to allow plaque center formation. Then the cells were fixed in 3.7% formaldehyde and stained with 1% crystal violet. The viral titer was calculated as the number of plaque-forming units per milliliter.

### Real-time RT-PCR

After electroporation, cells were cultured at 37°C with 5% CO_2_. At 12, 24, and 48 hpt, both cell pellets and culture supernatants were collected. For RNA extraction, cells were washed with PBS, lysed in 500 μL TRIzol reagent, and stored at −80°C. Viral RNAs in the supernatant of the transfected cells and the intracellular total RNAs were extracted. The viral RNA levels were quantified as previously described (32).

### Viral NS2B/3 protease cleavage assay

pCAGGS-based NS2B/3 and Cpr-expressing plasmids were co-transfected into HEK293T cells in a 1:1 ratio by mass using Fugene HD (Promega) according to the manufacturer’s protocols. At 24 hpt, the cells were lysed with RIPA buffer (Beyotime). The samples were denatured in SDS loading buffer at 95°C for 15 min, subjected to SDS-PAGE electrophoresis, and transferred onto a PVDF membrane (Millipore). Then, the PVDF membrane was blocked with 5% skim milk for 2 h. After sequential incubation with primary and secondary antibodies, the signals were visualized with a chemiluminescence system (ChemiDoc, Bio-Rad).

### RNA pull-down assay

RNA pull-down assays were performed as described previously (33, 34). A DNA fragment encoding the T7 promoter, the WNV 5′ UTR, the first 99 bp of the capsid gene, and the 3′ UTR was amplified by PCR. The product was purified and used as a template for *in vitro* transcription using the MEGAscript Kit (Invitrogen); the RNA was randomly labeled using a biotin labeling kit (Roche) and subsequently purified by lithium chloride precipitation. The control RNA (biotin-Ctrl.RNA) was transcribed from the pTRI Xef plasmid using the MEGAscript Kit, following the same procedure as detailed above. HEK293T cells (~1 × 10⁷) were separately transfected with expression plasmids encoding HA-tagged capsid proteins of WNV, DENV-2, YFV, and TBEV, respectively. At 36 hpt, the cells were lysed in RIP buffer (25 mM Tris-HCl [pH 7.5], 150 mM KCl, 0.5 mM DTT, and 0.05% [vol/vol] NP-40). The lysates were incubated with 2 μg of biotinylated WNV RNA for 3 h and pulled down with 20 μL of streptavidin-conjugated magnetic beads (Beyotime) for 2 h. The beads were then washed gently four times with NT2 buffer (50 mM Tris-HCl [pH 7.0], 150 mM NaCl, 1 mM MgCl_2_, and 0.05% [vol/vol] NP-40) and once with NT2 high-salt buffer (50 mM Tris-HCl [pH 7.0], 150 mM NaCl, 1 mM MgCl_2_, 50 mM NaCl, and 0.05% [vol/vol] NP-40). Finally, the beads were resuspended, and the bound proteins were analyzed by Western blotting.

### Statistical analysis

All data were analyzed using GraphPad Prism 8.0.2 software and expressed as geometric mean (standard deviation). Statistical significance was assigned when *P* values were <0.05. Significant differences between groups were determined using a one-way or two-way analysis of variance.

## RESULTS

### Construction and characterization of chimeric WNV-DENV-2 capsid viruses

**Fig 1:**
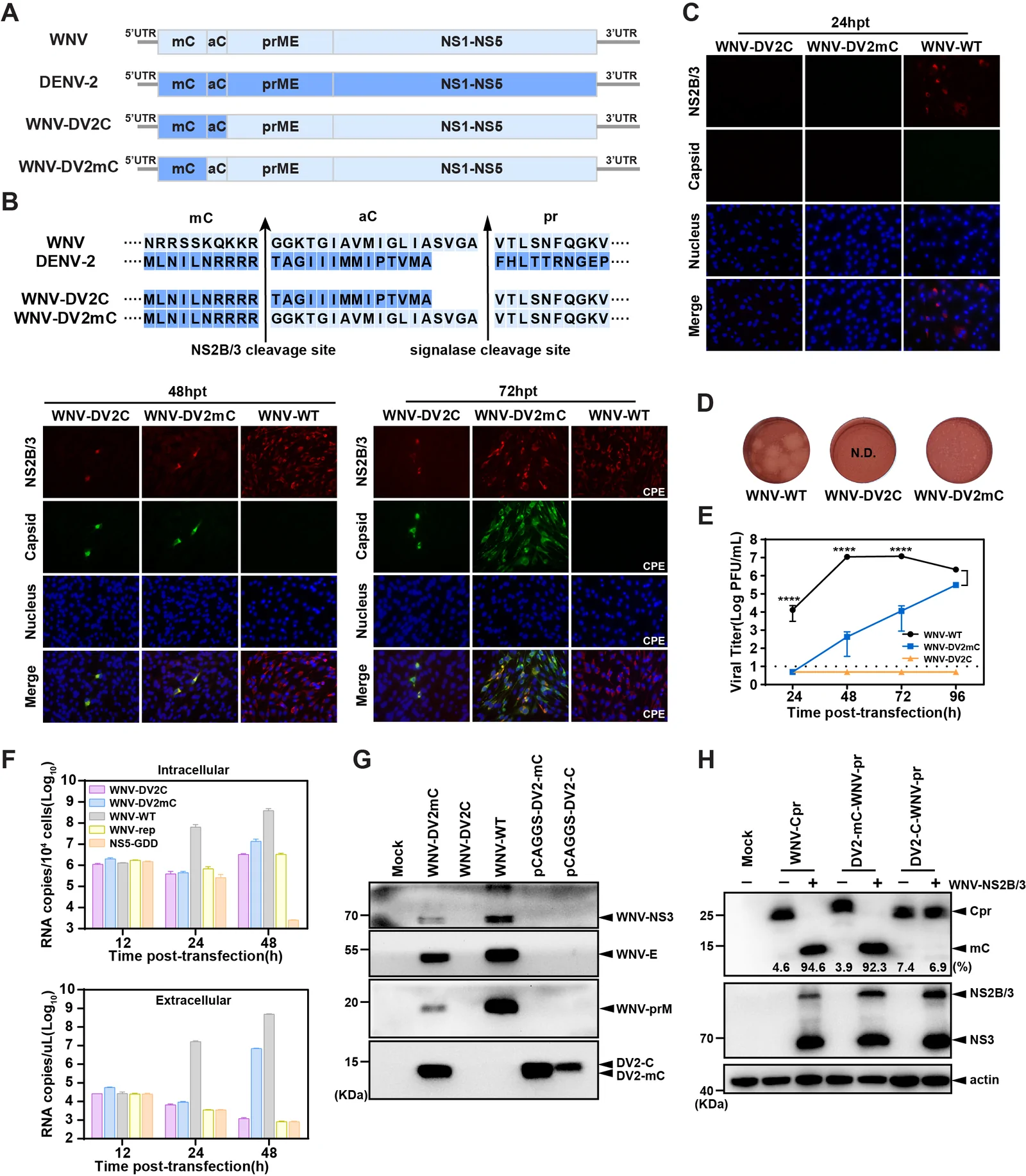
Generation and characterization of WNV and DENV-2 chimeric viruses. (**A**) Schematics of construction of WNV and DENV-2 chimeric viruses. aC, anchor capsid sequence (the short peptide of capsid C-terminal); mC, mature capsid sequence. Pale blue boxes indicate WNV genomes; light blue boxes indicate DENV-2 genomes. (**B**) Capsid and pr cleavage sites for WNV, DENV-2, and chimeric viruses. The NS2B/3 viral protease and signal peptidase cleavage sites are indicated by arrows. (**C**) IFA of RNA-transfected BHK-21 cells. Equal amounts of the *in vitro*-transcribed RNAs of WNV-DV2C, WNV-DV2mC, and WNV-WT were transfected into BHK-21 cells. At the indicated times post-transfection, DENV capsid and WNV NS2B/3 proteins were detected. (**D**) Comparison of plaque morphology. Plaque morphology of the chimeric virus and WNV-WT was compared by double-layer plaque assay. N.D., not detectable. (**E**) Virus growth kinetics. BHK-21 cells were transfected with equal amounts of the RNAs of WNV-WT, WNV-DV2C, and WNV-DV2mC. Extracellular infectious virions were quantified by plaque assay at 24, 48, 72, and 96 hpt. ****, *P* < 0.0001. (**F**) Copies of intracellular and extracellular viral RNAs. BHK-21 cells were electroporated with equal amounts of each RNA. Cell samples and culture supernatants were collected at the indicated time points. Total RNA or viral RNA was then extracted for subsequent quantification by qRT-PCR. (**G**) Viral protein processing in RNA-transfected BHK-21 cells. At 72 hpt, cells were harvested to detect viral protein expression, as well as to analyze the capsid protein and prME protein processing associated with WNV-DV2C and WNV-DV2mC. (**H**) Detection of WNV-NS2B/3 protease cleavage efficiency for chimeric structural proteins. HEK293T cells were transfected with plasmids encoding chimeric structural proteins or co-transfected with the pCAGGS-WNV-NS2B/3 plasmid. Cell lysates were harvested at 24 hpt and analyzed by SDS-PAGE and Western blotting. The cleavage efficiency of Cpr was assessed by immunoblotting with anti-His antibody. In parallel, the self-cleavage activity of NS2B/3 was monitored using anti-HA antibody.

To test the functional compatibility of heterologous capsid proteins, we first engineered WNV-DV2C by replacing the full WNV capsid with the DENV-2 capsid (Fig. 1A). This design was specifically chosen to investigate the structural and functional impacts of capsid protein exchange between these phylogenetically distinct orthoflaviviruses, which share only approximately 35% amino acid identity in their capsid proteins. After transfecting WNV-DV2C RNAs into BHK-21 cells, we observed minimal viral protein expression and no infectious particles in supernatants at 72 hpt (Fig. 1C), suggesting that full-length capsid replacement compromised both viral replication and packaging, and potentially impaired polyprotein processing by WNV NS2B/3 protease, likely due to incompatibility at the capsid–anchor junction.

To resolve this, WNV-DV2mC was constructed by retaining the WNV capsid anchor sequence (aC) along with the native WNV NS2B/3 protease cleavage sites while replacing the mature capsid (mC) with DENV-2 mC (Fig. 1B). Transfection of WNV-DV2mC RNAs into BHK-21 cells resulted in productive replication, with a concomitant increase in WNV-NS2B/3 and DENV-2 capsid protein expression observed from 24 to 72 hpt (Fig. 1C). However, WNV-DV2mC exhibited delayed replication compared to WNV-WT, as demonstrated by immunofluorescence assay (IFA) and growth kinetics (Fig. 1E). Moreover, WNV-DV2mC formed smaller plaques with fuzzy edges compared to WNV-WT (Fig. 1D).

To investigate the impact of capsid replacement on viral production, we quantified intracellular and extracellular RNA copy numbers of WNV-DV2mC, WNV-DV2C, and WNV-WT, alongside the WNV replicon as a control. Intracellular and extracellular RNA levels of WNV-DV2mC were significantly lower than those of WNV-WT; a modest increase was observed at 48 hpt. In contrast, WNV-DV2C replicated intracellularly at levels comparable to those of the WNV replicon, yet extracellular viral RNA was virtually undetectable (Fig. 1F). We then detected the polyprotein processing in the chimeric viruses-infected cells. While the CprME structural protein of WNV-DV2mC was efficiently cleaved to produce the mC, prM, and E proteins, processing of WNV-DV2C was severely impaired, with almost no detectable protein expression (Fig. 1G). Considering the essential role of sequential cleavage by NS2B/3 protease and host signal peptidase in virion morphogenesis (35, 36), we further investigated capsid protein processing efficiency. The C-pr expression plasmids were constructed for both WNV-DV2C and WNV-DV2mC (Fig. 1B), incorporating an N-terminal His tag to monitor cleavage products. Efficient processing would yield a 13 kDa His-tagged mC protein, distinct from the 25 kDa uncleaved C-pr precursor. In HEK293T cells co-transfected with the WNV-NS2B/3 and DV2-mC-WNV-pr plasmids, mC was detected at levels comparable to WNV-Cpr. In contrast, DV2-C-WNV-pr showed no detectable cleavage products (Fig. 1H).

These findings demonstrated that heterologous capsid protein can package WNV genomic RNA if polyprotein processing is preserved, albeit at a reduced virion production efficiency. The impaired viral yield observed in WNV-DV2mC may stem from (i) disrupted specific interactions between the DENV-2 capsid protein and WNV genomic RNA, (ii) altered *cis*-acting RNA sequences in the capsid-coding region that are essential for viral replication, or (iii) a combination of both mechanisms. To distinguish these possibilities, we serially passaged WNV-DV2mC in C6/36 cells and analyzed adaptive mutations.

### Serial viral passages of WNV-DV2mC identify compensatory mutations within the 5′ UTR (T78G) and capsid-coding region (A101C)

**Fig 2:**
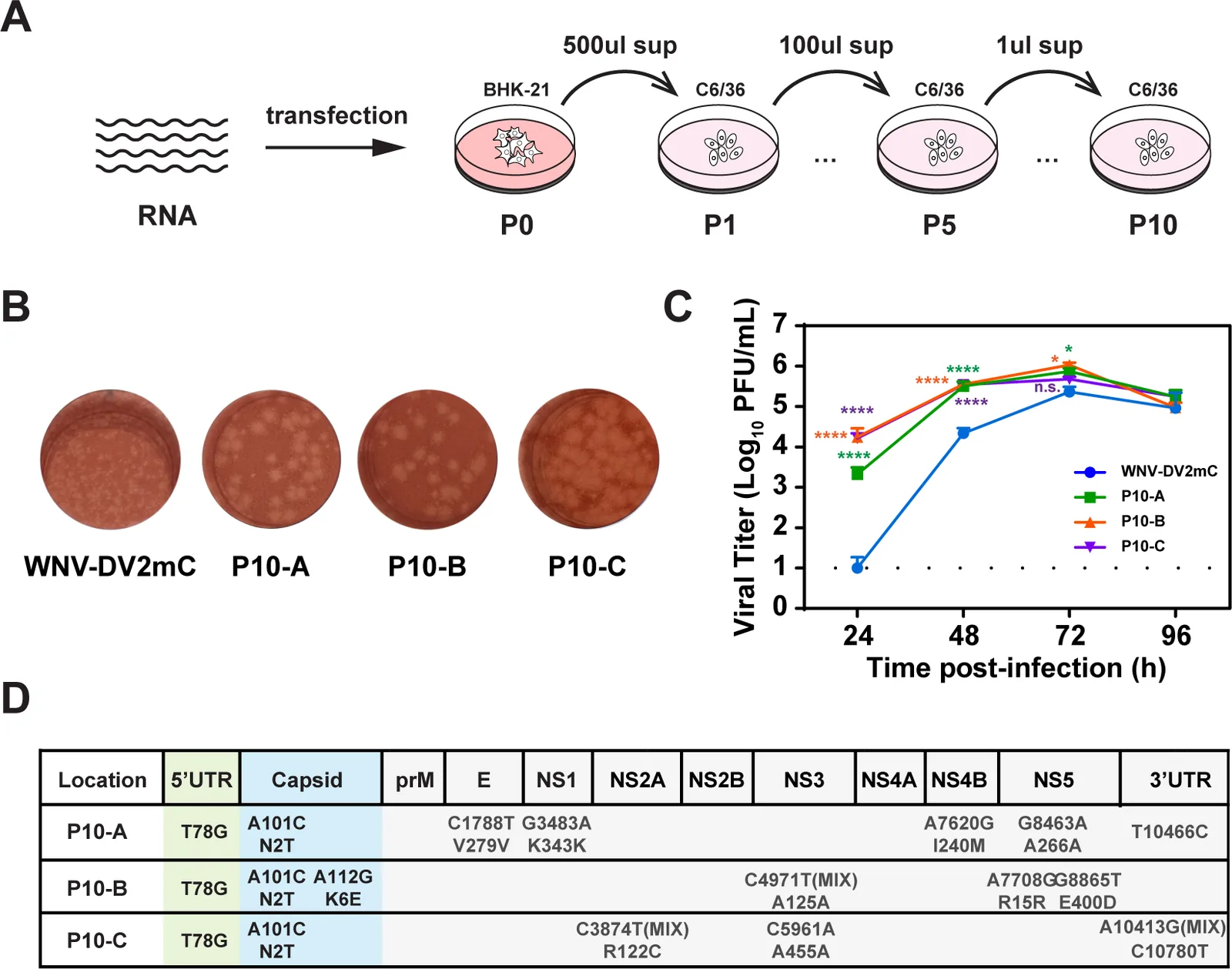
Characterization of passaged WNV-DV2mC mutants. (**A**) Schematic diagram of WNV-DV2mC virus passaging in C6/36 cells. (**B**) Plaque morphologies. Comparison of plaque sizes of the WNV-DV2mC P0 virus and P10 viruses. (**C**) Growth kinetics comparison. The WNV-DV2mC-P0 virus and WNV-DV2mC-P10-A/B/C viruses infected BHK-21 cells at an MOI of 0.1, respectively. Viral growth kinetics were determined by titrating the supernatants harvested at the indicated time points after infection using a plaque assay. n.s., no statistical significance. *, *P* < 0.1; ****, *P* < 0.0001. (**D**) Full-length genome sequencing and identification of adaptive mutations in the WNV-DV2mC-P10 passaged viruses. Intracellular viral RNAs were extracted from infected BHK-21 cells and subjected to RT-PCR, followed by cDNA sequencing. The corresponding amino acid and nucleotide sequences at each position are indicated.

WNV-DV2mC was subjected to serial passaging through three independent parallel lineages (Fig. 2A), yielding passage 10 (P10) supernatants (designated WNV-DV2mC-P10-A/B/C) that were used for subsequent phenotypic characterization. Plaque assay in BHK-21 cells revealed that WNV-DV2mC-P10-A/B/C produced larger, clearer plaques than P0 (Fig. 2B). Growth kinetic analysis demonstrated higher peak titers in P10 viruses compared to P0 (Fig. 2C), indicating successful recovery of viral production efficiency through serial passages.

**Fig 3:**
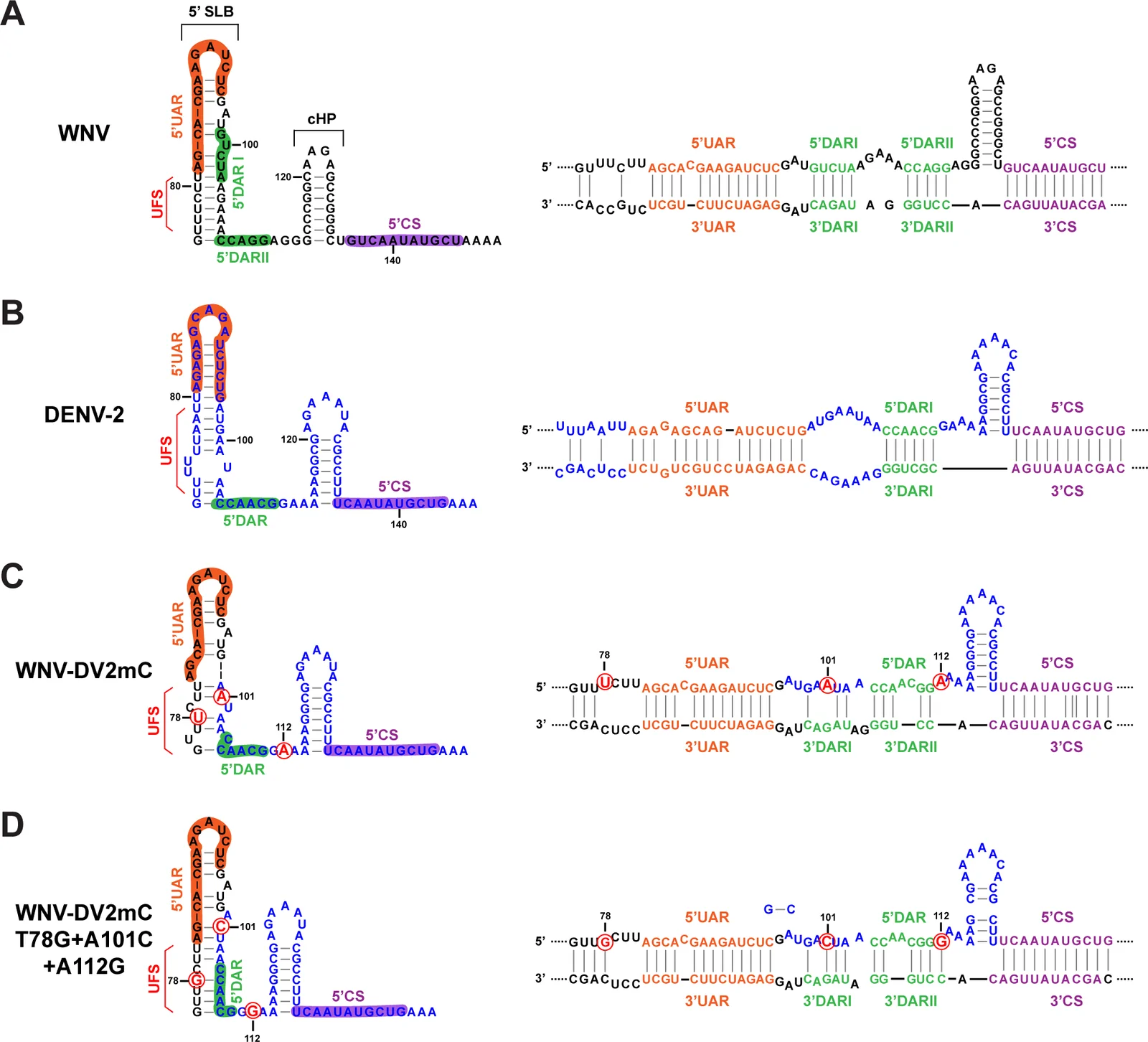
Diagram of the 5′ UTR structure and sequences of the mutant virus. The UAR, DAR, and CS elements are labeled in orange, green, and purple, respectively. (**A–D**) N-terminal secondary structure and long-range RNA–RNA interactions in WNV, DENV-2, WNV-DV2mC, and WNV-DV2mC mutant genomes. The WNV sequence is indicated in black, and the DENV-2 sequence is indicated in blue. The T78G, A101C, and A112G mutation sites are shown in red.

Whole-genome sequencing of P10 viruses identified two conserved mutations across all three lineages: T78G (5′ UTR) and A101C (capsid-coding region) (Fig. 2D). Considering the critical role of capsid-coding sequences, we performed comparative structural analysis using Mfold prediction (37) to predict RNA structure and found that the original capsid replacement disrupted essential base pairing between the 5′ UTR and 3′ UTR in WNV-DV2mC (Fig. 3A through C). The T78G and A101C mutations appear to restore these critical RNA interactions (Fig. 3D).

**Fig 4:**
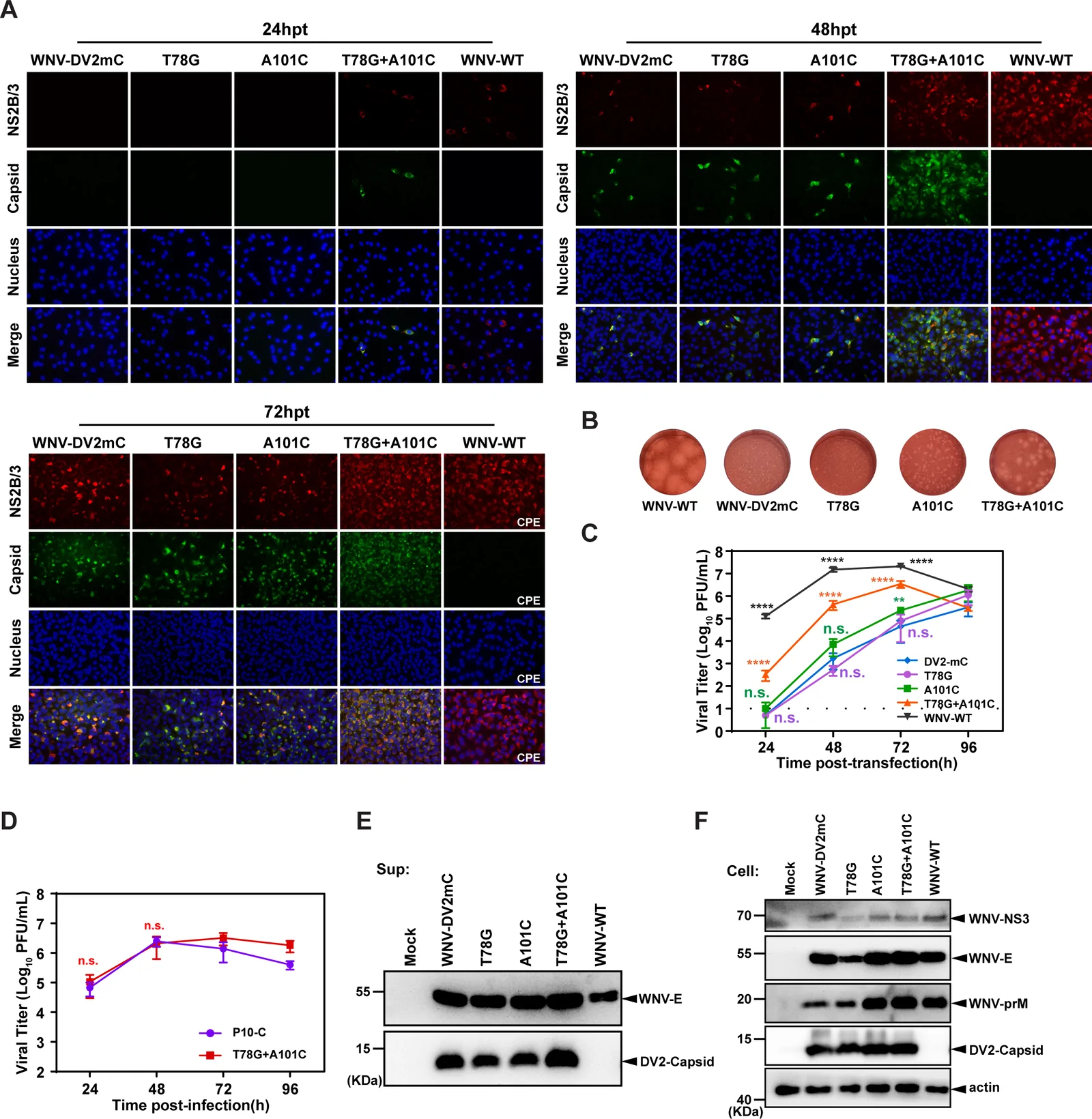
Characterization of adaptive mutations in WNV-DV2mC revertant viruses. (**A**) IFA of BHK-21 cells transfected with genome-length RNAs containing mutations. Equal amounts of mutant RNAs were transfected into BHK-21 cells, and IFA was performed at indicated time points post-transfection using polyclonal antibodies against DENV capsid and WNV-NS2B/3. (**B**) Plaque morphology. Culture supernatants from groups (**A**) were subjected to a double-layer plaque assay in BHK-21 cells. (**C and D**) Comparison of growth kinetics between the WNV-DV2mC virus, mutant viruses, and passaged virus in BHK-21 cells. Virus production in the supernatant was measured at indicated time points following transfection or infection. n.s., no statistical significance. **, *P* < 0.01; ****, *P* < 0.0001. (**E and F**) Virus protein detection. The viral structural proteins from mutant virus particles in the supernatant (E) and its expression/processing in infected cells (F) were analyzed by SDS-PAGE and Western blotting.

To validate the role of T78G and A101C mutations in restoring WNV-DV2mC viral production, we engineered WNV-DV2mC mutants carrying T78G alone, A101C alone, or T78G + A101C. Transfection of these RNAs into BHK-21 cells enabled simultaneous monitoring of viral production through IFA and viral titers at multiple time points. The double mutant T78G + A101C demonstrated significantly enhanced viral production efficiency, achieving 100% IFA-positive cells by 72 hpt and producing larger, more distinct plaques compared to WNV-DV2mC-P0 (Fig. 4A and B). Single mutants (T78G or A101C) exhibited limited viral production similar to WNV-DV2mC-P0. Our comparative growth kinetics analysis delineated distinct functional roles for these mutations. While T78G alone had minimal influence on viral production, A101C partially restored, and T78G + A101C achieved complete recovery, matching the efficiency of WNV-DV2mC-P10 (Fig. 4C and D). Moreover, WNV-E and DENV-C proteins were consistently detected in the supernatants of all mutant viruses (Fig. 4E), and correct processing of the C-prM-E protein was also successfully observed in virus-infected cells (Fig. 4F). These results demonstrate that the synergistic interaction between T78G and A101C mutations is essential for full restoration of WNV-DV2mC viral production efficiency.

### T78G and A101C mutations primarily enhanced viral production efficiency by augmenting viral genome replication

**Fig 5:**
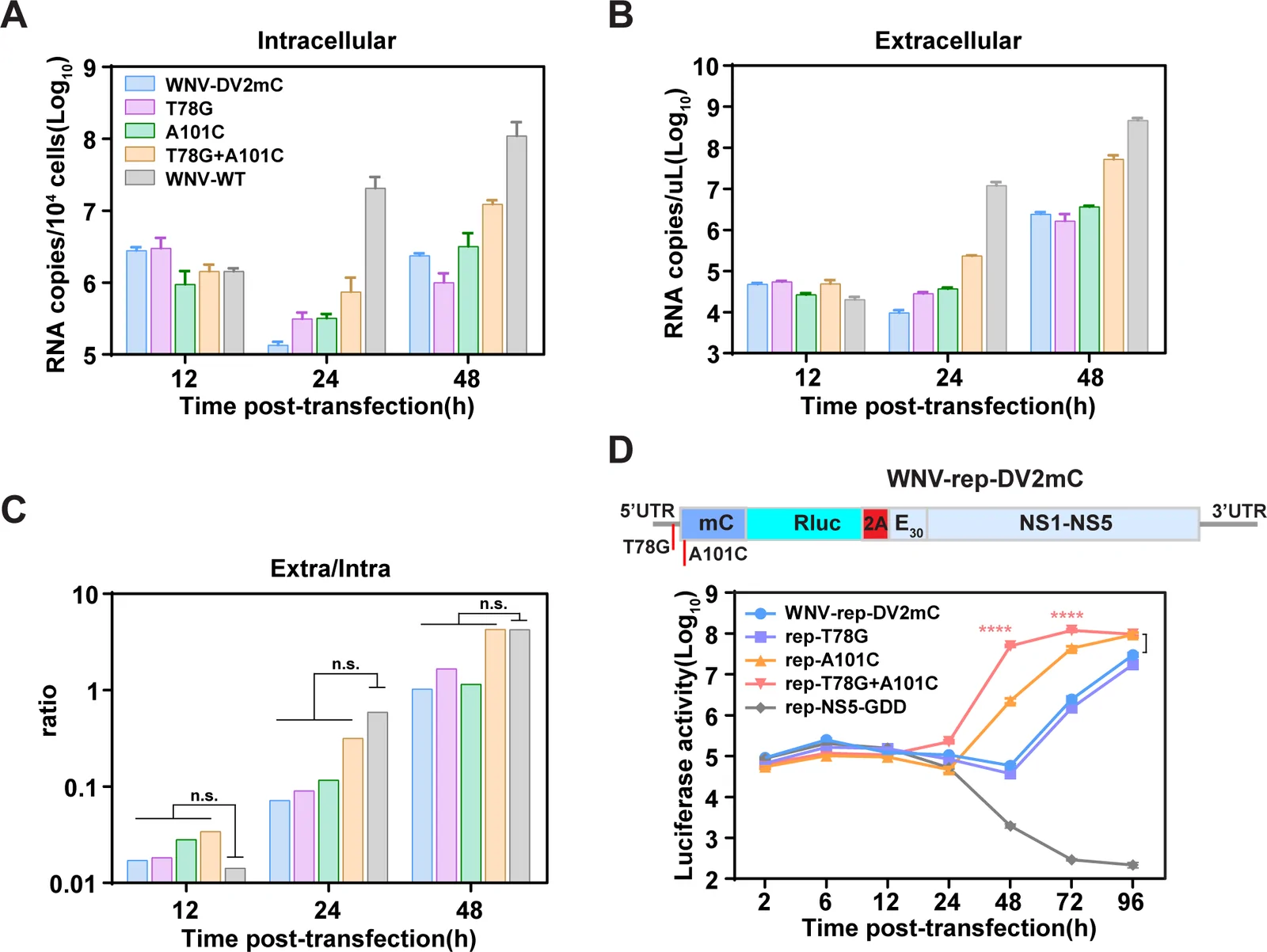
Analysis of adaptive mutations in replication and assembly/release. (**A and B**) Detection of viral RNA copies. BHK-21 cells were electroporated with WNV-DV2mC, mutant viruses, or WNV-WT RNAs. Cell samples (**A**) and culture supernatants (**B**) were collected, and RNA was extracted for subsequent quantification by qRT-PCR. (**C**) Ratios of extracellular/intracellular RNAs. The ratios were calculated by dividing the total amount of extracellular viral RNA by the total amount of intracellular viral RNA. n.s., no statistical significance. (**D**) Translation and replication of WNV-rep-DV2mC mutants in BHK-21 cells. Construction of the WNV-rep-DV2mC *Renilla* luciferase (Rluc) replicon (top panel). Equal amounts of WT and mutant RNAs were electroporated into BHK-21 cells. Subsequently, cell samples were collected to measure signal values (bottom panel). The means and standard deviations from three independent experiments are presented. ****, *P* < 0.0001.

To assess the impact of each mutation on viral RNA replication and virion production, we quantified intracellular and extracellular viral RNA levels. Across all WNV-DV2mC mutants, intracellular RNA copy numbers strongly correlated with their extracellular counterparts, consistent with the infectivity profiles shown in Fig. 4C, and this correlation indicates that the mutation does not affect viral assembly or release. The combined T78G and A101C mutations confer a moderate enhancement in the intracellular replication capacity of the WNV-DV2mC virus; however, its replication level remains lower than that of WNV-WT (Fig. 5A through C).

To further elucidate the effect of the mutations on viral translation and RNA synthesis, we used a chimeric WNV-rep-DV2mC luciferase replicon system (Fig. 5D, top panel) and the NS5-GDD replication-deficient replicon as a negative control. Transfection of BHK-21 cells with mutant replicons and subsequent luciferase measurements revealed comparable translation efficiency across all constructs at 6 hpt, demonstrating no effect on translation. However, RNA replication efficiency after 24 hpt showed distinct patterns: T78G alone sustained replication at a level comparable to that of WNV-rep-DV2mC; A101C moderately enhanced replication; and T78G + A101C exhibited significant improvement in replication efficiency (Fig. 5D, bottom panel). Our findings demonstrate that the adaptive mutations T78G and A101C, acquired in the 5′ UTR and capsid region during serial passages, primarily restore WNV-DV2mC replication through enhanced genome synthesis. RNA structural analysis revealed that these mutations facilitate viral replication by restoring base-pairing interactions in the UFS element and cyclization sequence (Fig. 3D). Importantly, *in vitro* cleavage assays confirmed that WNV-NS2B/3 protease efficiently processes the DV2-mC-WNV-pr, indicating proper capsid maturation and packaging functionality. These results collectively demonstrate that orthoflavivirus capsid proteins can package heterologous genomic RNAs when two critical conditions are met: (i) efficient genome replication and (ii) precise polyprotein processing.

### Orthoflavivirus capsid proteins mediate non-species-specific genome packaging

**Fig 6:**
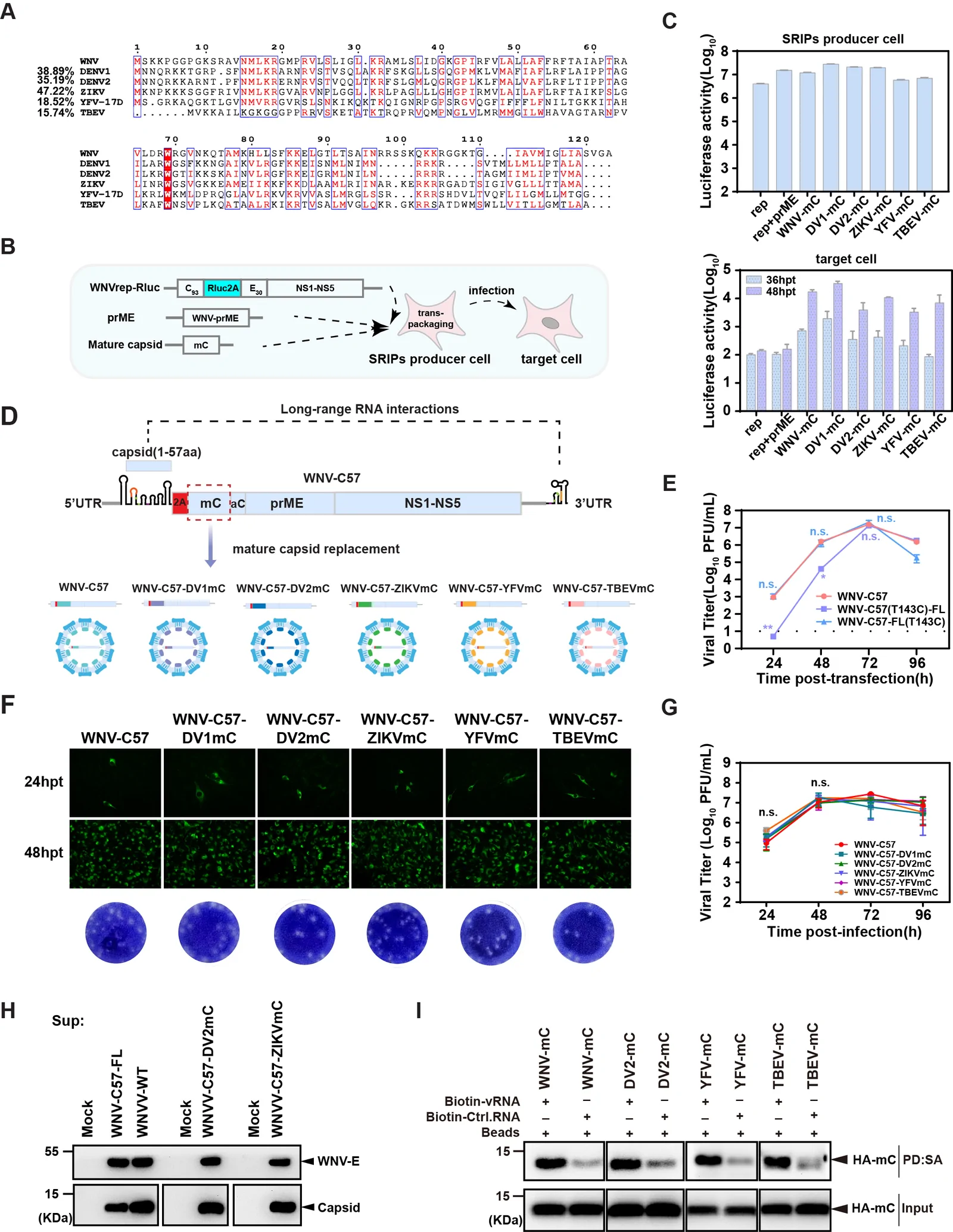
Assembly of SRIPs and characterization of WNV chimeric viruses with duplicated capsid sequences. (**A**) Multiple sequence alignment of orthoflavivirus capsid protein sequences generated by ClustalW. The percentages shown in the figure represent the sequence identity of each sequence compared with the WNV sequence. (**B and C**) Design of the SRIP-producing system and packaging of WNV replicon with WNV prME by different capsid proteins. (**B**) The WNV-Rluc2A replicon contains an ORF encoding an Rluc reporter gene fused to non-structural proteins via a 2A “self-cleaving” peptide. The prME and mC structural proteins were expressed using VEEV vectors, respectively. (**C**) Rluc levels in BHK-21 cells were quantified to estimate transfection efficiency (top panel). Subsequently, intracellular Rluc activity in the target cells was measured to estimate the amount of SRIPs in the producer cell supernatant (bottom panel). (**D**) Schematic representation of the WNV-C57 infectious clone, which was utilized to construct a panel of viruses with chimeric capsid sequences. The first 57 amino acids of the capsid protein (C57) were duplicated, as they encompass *cis*-acting elements critical for WNV RNA replication. These sequences were fused with the FMDV 2A sequence to generate the authentic N-terminus of the capsid protein. (**E**) Growth kinetics of WNV-C57 mutants. WNV-C57(T143C)-FL carries the T143C mutation in the C57 region downstream of the 5′ UTR, while WNV-C57-FL(T143C) carries the same mutation in the capsid-coding region. *, *P* < 0.1; **, *P* < 0.01. (**F**) IFA and plaque morphology of WNV-C57 chimeric viruses. Each of the chimeric viral RNAs shown in the figure (1 μg each) was transfected into BHK-21 cells. At 24 and 48 hpt, IFA was performed using anti-WNV-NS2B/3 antibody. At 72 hpt, the supernatants were collected, and viral plaque morphology was examined by the monolayer plaque assay. (**G**) Growth curves of recovered chimeric viruses in BHK-21 cells. BHK-21 cells were infected with the indicated viruses at an MOI of 0.1, and supernatants were collected for determination of virus titers by plaque assay. (**H**) Protein detection in WNV-C57-DV2mC and WNV-C57-ZIKVmC chimeric virions. The viral supernatant was analyzed by immunoblotting with antibodies against the WNV envelope protein and the capsid proteins of WNV, DENV-2, and ZIKV. (**I**) *In vitro* RNA-binding activities of purified capsid proteins from WNV, DENV-2, YFV, and TBEV. Biotinylated WNV RNA (Biotin-vRNA) and the negative control RNA (Biotin-Ctrl.RNA) were individually incubated with each recombinant capsid protein, followed by streptavidin pull-down assays (PD:SA). Bound proteins were detected by immunoblotting (IB) with an anti-HA antibody, and input levels of each capsid protein were similarly assessed by anti-HA IB.

To further validate the non-specific nature of orthoflavivirus capsid-mediated genome packaging, we first employed an SRIP production assay. This involved co-transfection of WNV replicon RNA (WNV-Rluc2A) and helper RNAs expressing different capsid proteins and WNV envelope protein (WNV-prME) (Fig. 6A and B). Expressing capsid proteins are selected from five representative orthoflaviviruses (DENV-1, DENV-2, ZIKV, YFV-17D, and TBEV). The packaging ability of genomic RNA by heterologous capsid was quantified by measuring Rluc activities after infection. Replicon signals in packaging cells showed no significant variation across groups, implying comparable transfection efficiencies (Fig. 6C, top panel). All tested SRIPs displayed comparable luciferase activities, indicating that heterologous capsids package WNV replicon RNA efficiently (Fig. 6C, bottom panel).

To eliminate cis-acting sequence effects, we engineered a WNV-C57 infectious clone (Fig. 6D) featuring (i) duplication of the RNA sequence encoding the first 57 amino acids of the WNV capsid, a region containing RNA elements critical for replication; (ii) incorporation of the FMDV 2A peptide to mediate cleavage and release of this duplicated 57-amino acid capsid fragment; and (iii) retention of the complete C-prM-E structural proteins and all non-structural proteins. Within the C57 region or full-length capsid-coding region, we introduced a single-nucleotide substitution, T143C, within the 5′CS, leading to a mismatch between 5′ and 3′CS. The WNV-C57(T143C)-FL and WNV-C57-FL(T143C) infectious clones were constructed, and their replication levels in transfected cells were compared. As shown in Fig. 6E, viral titers of WNV-C57(T143C)-FL were reduced, whereas WNV-C57-FL(T143C) exhibited a replication pattern similar to that of WNV-C57, indicating that the C57-derived sequence functions as a *cis*-element to support viral replication of WNV-C57 virus.

Subsequently, using WNV-C57 as the backbone, we generated a panel of chimeric viruses by replacing the mature capsid with DENV-1, DENV-2, ZIKV, YFV-17D, TBEV mC (Fig. 6D). These constructs retained all WNV-specific *cis*-acting replication elements and NS2B/3 protease cleavage sites. Recombinant chimeric viruses were successfully recovered in BHK-21 cells (Fig. 6F), and growth kinetics assays revealed that all chimeric viruses produced viral particles with efficiency comparable to that of WNV-C57 (Fig. 6G). Western blot analysis of virions further confirmed the incorporation of heterologous capsid proteins: both WNV-C57-DV2mC and WNV-C57-ZIKVmC virions contained the respective DENV-2 or ZIKV capsid protein (Fig. 6H).

To further characterize the capsid protein–RNA interaction, we examined the RNA-binding abilities of capsid proteins from WNV, DENV-2, YFV, and TBEV. Among these, the DENV-2 capsid protein shares relatively higher sequence homology with WNV, whereas YFV and TBEV exhibit lower sequence homology. Purified proteins were incubated with a biotinylated WNV ssRNA substrate or a control RNA (negative control). After pull-down with streptavidin-conjugated beads, the samples were resolved by SDS-PAGE to detect capsid-RNA interactions. All three heterologous capsid proteins exhibited efficient binding to WNV genomic RNA compared with the control RNA (Fig. 6I). These results provide compelling evidence that the capsid proteins of multiple orthoflavivirus species retain the ability to package the WNV genome into infectious virions when critical replication and processing elements are preserved. These findings indicate that orthoflavivirus capsid proteins are functionally interchangeable for genome packaging, supporting non-sequence-specific interactions.

## DISCUSSION

Nucleocapsid formation is a critical step in viral assembly. In viruses like HIV and HCV, specific packaging signals (HIV ψ element [38, 39] and HCV 3′ X-tail [40]) mediate selective RNA encapsidation. These regions are not only highly conserved but also harbor structured elements. Orthoflaviviruses, in particular, possess conserved RNA structures within their 5′ UTR, 3′ UTR, and capsid-coding region (41), suggesting the potential existence of RNA packaging motifs that may guide nucleocapsid assembly. However, no definitive capsid-RNA recognition sequences have been identified in orthoflavivirus. Here, we used chimeric viruses to systematically investigate capsid–genome interactions in orthoflaviviruses. Through the construction of chimeric viruses by replacing WNV capsid with DENV counterparts, we aimed to determine whether orthoflavivirus capsid proteins exhibit RNA packaging specificity or function through a more generic, sequence-independent manner.

The initial WNV-DV2C construct failed due to impaired processing of capsid proteins (Fig. 1C, E, and H), which is consistent with prior studies indicating that the P1′ site at the capsid–anchor junction is critical for NS2B/3 cleavage (42). Retaining the WNV anchor in the WNV-DV2mC restored processing and replication, albeit with reduced efficiency (Fig. 1C through F). Serial passaging led to the selection of compensatory mutations (T78G and A101C) in the UFS regions. Notably, the virus exhibited an additional A112G mutation in the capsid protein. Given that Zika virus replication requires stringent DAR complementarity (41) and considering that A112, located in the DARII region, is relatively conserved with the ZIKV DAR region, this mutation may exhibit a temperature-dependent effect. These mutations restored RNA structure and replication capacity by optimizing genome cyclization and enhancing RNA synthesis efficiency (Fig. 3 to 5). On the one hand, the T78G + A101C mutant virus exhibited a significant enhancement in virus replication efficiency. Consistent with this observation, the mutant virus produced substantially larger plaques than WNV-DV2mC, likely attributable to improved viral propagation resulting from elevated genomic RNA replication levels, which in turn influenced plaque morphology. On the other hand, both the growth kinetics and plaque size of the mutant virus remained lower than those of the WNV-WT. We hypothesize that although serial passaging selected for an adaptive mutation restoring base pairing within the UFS, such restoration conferred only partial functional recovery. Alternatively, additional, yet unidentified mechanisms of attenuation effects in capsid chimeric orthoflaviviruses, analogous to those observed in prME chimeric viruses, may contribute to the attenuated phenotype (43–45). These findings may provide new insights for the future design of attenuated orthoflavivirus vaccine candidates. Collectively, this evolutionary trajectory suggests that the observed replication defect primarily stems from RNA structural perturbations affecting replication rather than impaired nucleocapsid assembly.

To determine whether capsid proteins from other orthoflaviviruses within the same genus are functional in a heterologous context, we performed SRIP production assay to test their ability to package the WNV genome and assemble into infectious particles. Our results indicate that capsid proteins from different orthoflaviviruses are capable of efficiently packaging the WNV genome and assembling infectious viral particles (Fig. 6C). However, minor variations in assembly efficiency were observed among the different capsid proteins, suggesting the possible involvement of yet unidentified packaging constraints or regulatory mechanisms. Moreover, by using WNV-C57 chimeras, we demonstrated that capsids derived from DENV-1, DENV-2, ZIKV, YFV-17D, and TBEV efficiently package WNV genomic RNA (Fig. 6F through H). This strategy preserved the native context of viral replication and assembly, enabling a direct evaluation of packaging efficiency and specificity in infectious virus production, overcoming limitations of previous *in vitro* or VLP systems. This interchangeability suggests that packaging is mediated by conserved structural features rather than sequence-specific interactions. However, results from some studies indicate that orthoflavivirus capsid proteins are incapable of efficiently packaging heterologous RNA, which may be attributed to the lack of consideration for the NS2B/3 protease processing requirement within the capsid protein sequence (46).

To investigate the direct binding of heterologous orthoflavivirus capsid proteins to WNV genomic RNA, we performed an RNA pull-down assay. In this experiment, we selected capsid proteins from DENV-2, YFV, and TBEV as representative orthoflaviviruses exhibiting varying degrees of amino acid sequence identity with the WNV capsid protein, with DENV-2 showing the highest homology (35%), followed by YFV (18%) and TBEV (15%). Consistent with our prior observations, all three heterologous capsid proteins exhibited efficient binding to WNV genomic RNA (Fig. 6I). The results further confirmed the RNA-binding properties of orthoflavivirus capsid proteins.

These findings hold significant implications for both orthoflavivirus biology and vaccine development. The demonstrated capsid interchangeability provides a valuable tool for chimeric vaccine design, while the potential existence of specific packaging signals offers new targets for antiviral strategies. Future studies employing cryo-EM structural analysis, deep mutational scanning, and single-molecule packaging assays will be crucial for further elucidating the sophisticated balance between conserved packaging mechanisms and virus-specific adaptations that govern orthoflavivirus assembly.

## Data Availability

All data supporting the findings of this study are available within the article or from the corresponding author upon reasonable request.
